# The Impact of an Experiential Social Medicine Curriculum in an Emergency Medicine Residency Training Program: Mixed-methods Curricular Evaluation

**DOI:** 10.5811/westjem.2022.10.57724

**Published:** 2022-12-29

**Authors:** Hurnan Vongsachang, Todd Schneberk, Laura Sprunt, Gabe Padilla, Jeff Riddell

**Affiliations:** * Los Angeles County + University of Southern California Medical Center, Department of Emergency Medicine, Los Angeles, California; † Temple University Hospital, Department of Emergency Medicine, Philadelphia, Pennsylvania; ‡ Rhode Island Hospital and Brown Emergency Medicine Residency, Providence, Rhode Island; § Keck School of Medicine of the University of Southern California, Department of Emergency Medicine, Los Angeles, California

## BACKGROUND

Social medicine (SM) is an emerging field that includes the study of the social determinants of health (SDoH), which include addressing health disparities, cultural competency, service, and population health.^1^,^2^ Despite widespread acknowledgment of its influence in patient care, SM is underemphasized in graduate medical education.^3^–^6^ There have been numerous attempts to incorporate SM into both medical school and residency curricula in existing literature, most of which suggest promising results especially when learning is experiential in nature.^2^,^7^–^17^ However, there is still no widely accepted or standardized experiential SM curriculum in postgraduate training.^18^–^20^

As the scope and practice of emergency medicine (EM) is intimately tied with SM, there has been a recent focus on incorporating SM curricula into existing residency programs to better care for the underserved patients who often rely on the emergency department (ED).^21^,^22^ A limited number of EM residency programs have developed and/or integrated a SM curriculum into their training programs; however, most are didactic-based and have varying experiential components.^23^–^25^ Despite calls for formal evaluation of SM curricula, the impact of experiential SM curricula on EM residents’ attitudes and behaviors remains unclear. Developing and evaluating an SM curriculum can address this gap and improve residents’ attitudes toward, understanding of, and perceived ability to care for vulnerable patients who often seek care in the ED.

## OBJECTIVES

To address this gap, we aimed to develop and evaluate an SM elective where EM residents learn from experience. We sought to understand the impact of this type of curriculum on residents’ attitudes toward and self-reported ability to care for vulnerable populations.

## CURRICULAR DESIGN

This study received approval from the institutional review board. From July 2018–2019, all 73 residents at our EM residency program were invited to participate in experiential two-week electives focused on patients from seven vulnerable subpopulations: persons experiencing substance use disorders; experiencing homelessness; having been seen at a border health clinic; seeking asylum, facing primary care access barriers; having been involved in the Violence Intervention Program (VIP), or involved with the carceral system. Participation was voluntary. Experiences were coordinated with community-based organizations (CBO) and tailored to the resident’s interest and prior exposure.

The SM curriculum was developed by a task force of ED faculty, residents, community-engaged faculty, and linked CBOs that were active in addressing SDoH from the ED in a multidisciplinary perspective. Partnerships were built on prior connections with CBOs and existing linkages between the department and the community. Faculty reached out to CBOs already interacting with ED care, such as on-campus VIPs, and established more robust and defined routes of resident involvement in programming for a two-week elective period. Care was taken in working with CBOs to ensure that resident involvement would not be onerous to their staff or disrupt workflow and that the task force incorporated an approach beneficial to their service delivery whenever possible. Content expert faculty who helped with development of electives included the director of the street medicine program, sociology research faculty working with vulnerable populations in needle-exchange settings, the director of addiction medicine for the medical center, the faculty directors of the Keck University of Southern California Human Rights Asylum Clinic, the director of the urgent care clinic and outpatient clinic care coordination, and a trauma surgery faculty member who serves on the board of the violence intervention CBO. The main faculty member developing and coordinating the curriculum development (TS) was fellowship trained in community-based research methods and SM, and has experience with a previous SM curriculum development at another program.

In addition to resident educational experience, the task force valued solidarity with CBOs and benefit to patients as primary goals. The curriculum was driven by CBOs that were activated, interested, and willing to provide learning opportunities for residents. We recognized that residents were present to learn from the CBOs; thus, the intention was to not overwhelm CBOs with extra tasks. Finally, we also favored CBOs who had already been involved in providing services in the ED with the intention of maximizing the overlap of patients between CBOs and the ED.

Objectives were to expose residents to CBOs working with these specific populations with the goal of better understanding the structural vulnerability of these populations. These rotations exposed residents to services provided by the CBO and how those services can be incorporated into the care of these patients in the ED. For example, for VIP, residents observed and assisted the community partner with service provision including job placement, peer counseling, and tattoo removal, and subsequently learned how to better incorporate referrals during routine ED care. This strategy used the expertise of the CBO while extending ED care to incorporate a community perspective. Sample activities from each experience are listed in Table 1. We recognize that there is heterogeneity across CBOs and the overlap with care delivery in the ED is not uniform. Therefore, we did not develop or enforce experience-specific resident evaluations.

## IMPACT AND EFFECTIVENESS

We invited participants to complete a voluntary, anonymous, post-rotation electronic survey exploring changes in their attitudes and competence (Appendix 1). Items were adapted from existing surveys on attitude change in public health and SM, and pilot tested with non-involved residents of the curriculum.^26^,^27^ No changes were made to the survey based on the pilot results. In the year following completion of the SM elective, we also conducted semi-structured interviews with a convenience sample of seven participants to explore a deeper understanding of the SM experience and provide a rich description of how it impacted them. Interviews were conducted in delayed fashion to explore any sustained impacts of the experience. Interview questions aimed to explore residents’ self-reported changes in attitudes toward and behaviors in caring for vulnerable populations. Interviews were audio-recorded, de-identified, and transcribed. Two authors (HV, LS) performed a reflexive thematic analysis of resulting transcripts.^28^ They familiarized themselves with the data, generated initial codes, searched, reviewed and defined themes, and wrote up the results of the analysis.^29^

Of the 38 residents who participated, 22 completed the survey (58%). Participants reported increased understanding, satisfaction, empathy, perceived responsibility, and perceived competence towards working with vulnerable populations after their elective (Table 2a). Any references to behavior change are self-reported from survey data, which has shown validity for behavior change in other medical education contexts.^30^ However, given that we did not directly measure changes in clinical care, we attributed all references to behavior change in the context of self-reported changes. Both patient- and resident-oriented themes were identified in the interviews (Table 2b).

First, participants reported increased understanding of the healthcare challenges faced by vulnerable populations. One participant offered that their carceral health elective:

…definitely narrowed the gap in knowledge, significantly, as far as understanding their experience in the jails. I got to go to this space where my patients come to me from. How often do you ever get to do that and understand their perspectives and their experience from like directly going into the place…and seeing it? (Participant #5)

Participants commented on the increased sense of empathy that came from their experience. Participants also reported perceived increased confidence and clinical competence when caring for these patients, as the experiences “made it easier to come back and work in the setting that I was working in and be able to bring lessons from both to each place.” (Participant #7) Participants noted that the elective provided a sense of rejuvenation, as “it re-inspired some of those folks or at least made those conversations a bigger part of every shift.” (Participant #7) The SM elective also was integral in career development. One participant voiced, “I’d like to be involved in health system development or community outreach or something at least part time for my career.” (Participant #2)

Finally, participants offered suggestions for future iterations of the elective, such as a hybrid curriculum, including formalized didactic lectures. Most participants also requested a more longitudinal experience to “keep the momentum going longer.” (Participant #6) One participant also voiced that “a social EM, formalized curriculum should be a mandatory part of training in this program that happens early on in residency because I think it really does impact the type of care and follow-up that we provide to our patients.” (Participant #2)

## LIMITATIONS AND CONCLUSION

While the results of our SM curriculum suggest that it positively impacted residents’ attitudes and informed their care of vulnerable populations, several limitations exist. Although the construct of partnership with CBOs is generalizable, the exact CBOs with which we worked vary geographically and demographically, which may limit reproducibility. Our innovation also involved a single institution and medical specialty, which may further limit its generalizability. Individuals who completed the surveys are susceptible to varying levels of response bias. Our qualitative findings are limited by the small sample size of resident interviews, whose voluntary participation may also introduce selection bias.

Despite these limitations, our experiential SM curriculum positively impacted residents’ attitudes and informed their care of vulnerable populations. It also empowered residents in addressing SDoH on shift. Given the pervasive impact of the SDoH in the practice of EM, it may be useful for residency program leaders to integrate experiential electives into residency curricula. Future research may include community-based participatory research methods with existing CBOs to understand the perceived attitudes, challenges, and opportunities that CBOs have in facilitating and receiving hand-off of patients from clinical providers. Resident practice patterns in referring and linking patients to care beyond the ED should also be examined as well. Resident performance milestones consistent with current Accreditation Council for Graduate Medical Education guidelines (ie, quality improvement, system navigation for patient-centered care, physician role in healthcare systems under “systems-based practice”; as well as practice-based learning, professionalism and interpersonal and communication skills) could also be examined in existing residency-specific evaluation avenues. Finally, as this was a preliminary and foundational study, patient-centered outcomes were not studied and are important to examine in future iterations.

## Supplementary Information



## Figures and Tables

**Table 1:** Description of Social Medicine Elective Experiences

Elective experience	Sample Activities
Patients with substance use disorders	Inpatient: respond to and see consults with the Addiction Medicine team to provide recommendations for drug use treatment ED: Review in real-time cases related to drug abuse or overdose with the Addiction Medicine team; speak with patients regarding drug use behaviors and readiness for change. If patient expresses interest in cessation, provide provide recommendations for drug use cessation For all patients seen by Addiction Medicine team and who have expressed interest, provide follow up appointment prior to discharge to ensure continuity of care Develop materials to guide harm reduction practices in the ED and resident education regarding concepts such as motivational interviewing and reducing patient stigma
Patient experiencing homelessness	Attend “Street Rounds” with the Street Medicine team to provide local wound care and supplies for patients Observe practice patterns of street medicine team and work with them to develop strategies for improvement of ED referrals and optimize ED consults to their service
Patients seen at border health clinic	Provide local wound care and medical consultations under attending supervision Provide harm reduction services such as naloxone, fentanyl test strips, hygiene kits, drug use supplies, and patient education about said materials Learn about intersection of immigration and drug use in a trans-national setting and the specific local barriers to care and harm reduction ideology
Patient seeking asylum	Receive training on writing medical evaluations for patients seeking asylum Participate in and document medical evaluations for patients seeking asylum under attending supervision Participate in avenues for client advocacy and learn from lawyers about the legal determinants of health regarding the asylum seeker population
Patient facing primary care access barriers	Engage in projects with hospital administration to improve upon the referral process for patients who are under- or uninsured Diagramming the local barriers to primary care access and subspecialty access within different types of insurance and work toward developing workflows for patients experiencing these access barriers
Patients involved in the Violence Intervention Program (VIP)	Observe and assist community partner with service provision including job placement, peer counseling, and tattoo removal through ED bedside engagement Learn how better to incorporate referrals during routine ED care and improve department-wide workflows with community-based organization
Patients involved with carceral system	Shadow and learn from current staff, providers, and patients at the county jail to better understand logistics (i.e. timing, equipment, staff, material resources) and other challenges involved in patient care Develop quality improvement initiatives for care transitions between providers in the county jail and ED care

**Table 2a:** Aggregate post-elective experience survey scores by domain.

Attitude domain #1 (N=22)					
Compared to how you felt prior to this elective, how would you rate your:	1 = Strongly Decreased	2 = Decreased	3 = Unchanged	4 = Increased	5 = Strongly Increased
Understanding of healthcare challenges faced by *?	0 (0%)	0 (0%)	0 (0%)	12 (54.5%)	10 (45.5%)
Ability to empathize with *?	0 (0%)	0 (0%)	0 (0%)	9 (40.9%)	13 (59.1%)
Sense of satisfaction when treating *?	0 (0%)	0 (0%)	0 (0%)	9 (40.9%)	13 (59.1%)
Sense of frustration when treating *?	0 (0%)	6 (27.2%)	9 (40.9%)	3 (13.6%)	4 (18.1%)

Attitude domain #2 (N=22)					

Compared to how you felt prior to this elective, how would you rate your level of agreement with the following statement:	1 = Strongly Disagree	2 = Disagree	3 = Neutral	4 = Agree	5 = Strongly Agree
Emergency physicians are responsible for identifying and intervening on social determinants of health for *.	0 (0%)	1 (4.5%)	1 (4.5%)	6 (27.2%)	14 (63.6%)
There is a LOT that I can do to help *in the emergency department.	0 (0%)	1 (4.5%)	4 (18.1%)	10 (45.5%)	7 (31.8%)

Competence domain (N=21)					

Compared to how you felt prior to this elective, how would you rate your:	1 = Strongly Decreased	2 = Decreased	3 = Unchanged	4 = Increased	5 = Strongly Increased
Knowledge of the social support services and/or resources available to * at our institution?	0 (0%)	0 (0%)	5 (23.8%)	10 (47.6%)	6 (28.6%)
Ability to identify the social determinants of health that are contributing to a(n) * presentation?	0 (0%)	0 (0%)	2 (9.5%)	13 (61.9%)	6 (28.6%)
Ability to establish a therapeutic alliance with *?	0 (0%)	0 (0%)	3 (14.3%)	11 (52.4%)	7 (33.3%)
Ability to intervene on the social issues that are contributing to a(n) * presentation?	0 (0%)	1 (4.8%)	5 (23.8%)	11 (52.4%)	4 (19.0%)

Data are reported n (%).

* Patients experiencing substance use disorders, experiencing homelessness, being seen at the border health clinic, seeking asylum, facing primary care access barriers, being involved in the Violence Intervention Program at our hospital, or being involved with the carceral system.

**Table 2b:** Continued. Themes from semi-structured interviews (N=7).

Themes	Supporting Quotes
1. Patient-oriented themes	
A. Residents reported a deeper understanding into the healthcare challenges faced by *.	“For the most part, it’s been helpful to just understand what the landscape looks like in the Los Angeles area and get a sense of that…Certainly having a better understanding of what community resources were out there and some of the challenges facing the LA population specifically was helpful because I have [now] a little more literacy to know what the landscape is when we have to refer out.” (Transcript 4)
B. Residents reported increased perceived confidence when caring for *.	“I think, especially for asylum work, it sounds really intimidating to be writing these affidavits and you’re doing these interviews, and it sounds (at least to me) really daunting - like “oh my god that’s intense, I don’t know if I’m cut out for that,” but then getting to see that this is a very achievable process and it’s not so bad.” (Transcript 1) “I think there were times that things felt comfortable or more comfortable or I felt more confident because people would come back and clearly have used the needles that we did give out for harm reduction or tell us about how they saved someone’s life with the Narcan.” (Transcript 7)
C. Residents reported increased perceived clinical competence when caring for *.	“I think it’s changed the way that I’m able to plug patients into care and safely discharge patients and kind of recognize needs, as well.” (Transcript 3) “Obviously we’re talking orders of magnitude difference, but I think the mindset of having to fight those fights a lot for patients really allows it or makes it easier to get involved with the Wound Clinic and also made it easier to come back and work in the setting that I was working in and be able to bring lessons from both to each place.” (Transcript 7)
D. Residents reported increased motivation when caring for *	“So, it’s been only positive I feel in terms of motivating on-shift compassion and feeling, you know, empowered to actually make a difference - even if it’s not supported from top down. I think there are a lot of us who care and who kind of share best practices with each other.” (Transcript 2)
E. Residents reported increased empathy when caring for *.	“But, you know, I think when you understand where people come from, where their backstory is, or why people go to Tijuana for random medications and things, or what Tijuana is actually like, what the healthcare system there is like, what access our undocumented patients have, or what issues and barriers they face, it makes you more compassionate when they show up to the emergency department because you understand how limited they are.” (Transcript 2)
F. Residents reported frustrations when caring for *.	“But I think it’s really frustrating when it feels like every day in the emergency department, I don’t get to be the doctor that I want to be because our current system and pathways just don’t care to address some of these issues that our patients face. So, I end up spending a lot of extra time, trying to find and print out resources for patients or trying to explain to patients the different opportunities that they have for healthcare or for follow-up or for legal services…It sucks to feel as though you are an actor in a system that doesn’t really serve our patients well. And it’s out of your control.” (Transcript 2) “Another hard thing was just the recognition that you can help, but you can’t fix everything, which is something that we are all aware of every day.” (Transcript 5)

2. Resident-oriented themes	

A. Residents reported that the elective helped career development.	“I think it’s maintained my interest in social emergency medicine. I think it’s actually made me a little bit more interested in administrative work as well, and using those two pathways to go down a joint mission together - to use systems to improve access to care and to improve social emergency care for patients.” (Transcript 3) “I was using [the elective] to confirm that I did want to stay involved in that space… it changed my career goals in that it just solidified that I do want to keep working with this patient population.” (Transcript 5)
B. Residents reported a sense of rejuvenation with the elective.	“It’s really inspired me to make sure I’m involved in that and really mindful about allocating time and energy for that in the future and funding if I can. It’s definitely made it something that I definitely want to be a part of more.” (Transcript 1) “I think it was a huge rallying point for a lot of folks. Also, I think it brought people out of the woodwork or back from the brink - people who might have been disillusioned or kind of lost sight of some of the reasons why they showed up to do the work every day. It re-inspired some of those folks or at least made those conversations a bigger part of every shift or allowed it to come back to spaces that might have been inhabited by complaining about schedules or talking about other things related to clinical work and residency.” (Transcript 7)
C. Residents enjoyed the sense of ownership with regard to curriculum development.	“I think the other thing that I didn’t bring up that’s really awesome to see over the last few years too, and it’s been kind of cool to see develop is how the curriculum has really been championed by the people who want to learn in that curriculum. I feel like a lot of the so-called learners or residents are the people who are designed to gain something by going through it have also added a lot to it. And so it’s been this very awesome reciprocal relationship that is just allowing it to snowball. It’s kind of like a really beautiful thing. I can’t really think of any other curriculum that I’ve been a part of, as a student that I’ve seen grow like that where people are really growing it from the bottom up as they’re partaking in it. It almost feels like a potluck in some ways, which is kind of cool.” (Transcript 7)

* Patients experiencing substance use disorders, experiencing homelessness, being seen at the border health clinic, seeking asylum, facing primary care access barriers, being involved in the Violence Intervention Program at our hospital, or being involved with the carceral system.
