# Supplementary material for: The Impact of an Experiential Social Medicine Curriculum in an Emergency Medicine Residency Training Program: Mixed-methods Curricular Evaluation
Source: West J Emerg Med. 2022 Dec 29;24(1):83–8. doi: 10.5811/westjem.2022.10.57724 (PMC9897255; doi:10.5811/westjem.2022.10.57724)
Supplement: Supplementary file 1 [file wjem-24-83-s001.docx]

Appendix 1.

**Perceived Attitudes**

| Compared to how you felt prior to this elective, how would you rate your: | 1 = Strongly Decreased | 2 = Decreased | 3 = Unchanged | 4 = Increased | 5 = Strongly Increased |
| --- | --- | --- | --- | --- | --- |
| Understanding of healthcare challenges faced by *? |  |  |  |  |  |
| Ability to empathize with *? |  |  |  |  |  |
| Sense of satisfaction when treating *? |  |  |  |  |  |
| Sense of frustration when treating *? |  |  |  |  |  |

**Perceived Responsibility**

| Compared to how you felt prior to this elective, how would you rate your level of agreement with the following statement: | 1 = Strongly Disagree | 2 = Disagree | 3 = Neutral | 4 = Agree | 5 = Strongly Agree |
| --- | --- | --- | --- | --- | --- |
| Emergency physicians are responsible for identifying and intervening on social determinants of health for *. |  |  |  |  |  |
| There is a LOT that I can do to help *in the emergency department. |  |  |  |  |  |

**Perceived Competence**

| Compared to how you felt prior to this elective, how would you rate your: | 1 = Strongly Decreased | 2 = Decreased | 3 = Unchanged | 4 = Increased | 5 = Strongly Increased |
| --- | --- | --- | --- | --- | --- |
| Knowledge of the social support services and/or resources available to * at our institution? |  |  |  |  |  |
| Ability to identify the social determinants of health that are contributing to a(n) * presentation? |  |  |  |  |  |
| Ability to establish a therapeutic alliance with *? |  |  |  |  |  |
| Ability to intervene on the social issues that are contributing to a(n) * presentation? |  |  |  |  |  |

**Patients experiencing substance use disorders, experiencing homelessness, seen at the border health clinic, seeking asylum, facing primary care access barriers, involved in the Violence Intervention Program (VIP) at our hospital, or involved with the carceral system.*
